# Influenza A(H7N9) Pandemic Preparedness: Assessment of the Breadth of Heterologous Antibody Responses to Emerging Viruses from Multiple Pre-Pandemic Vaccines and Population Immunity

**DOI:** 10.3390/vaccines10111856

**Published:** 2022-11-01

**Authors:** Min Z. Levine, Crystal Holiday, Yaohui Bai, Weimin Zhong, Feng Liu, Stacie Jefferson, F. Liaini Gross, Wen-pin Tzeng, Louis Fries, Gale Smith, Philippe Boutet, Damien Friel, Bruce L. Innis, Corey P. Mallett, C. Todd Davis, David E. Wentworth, Ian A. York, James Stevens, Jacqueline M. Katz, Terrence Tumpey

**Affiliations:** 1 Influenza Division, National Center for Immunization and Respiratory Diseases, Centers for Disease Control and Prevention, Atlanta, GA 30329, USA; 2 Novavax, Inc., Gaithersburg, MD 20878, USA; 3 GSK, 1300 Wavre, Belgium; 4 GSK, Rockville, MD 20850, USA

**Keywords:** A(H7N9) viruses, influenza vaccine, pandemic preparedness, population immunity, heterologous cross-reactivity, hemagglutination inhibition, neutralizing antibody, neuraminidase inhibition antibody, ADCC antibody

## Abstract

Influenza A(H7N9) viruses remain as a high pandemic threat. The continued evolution of the A(H7N9) viruses poses major challenges in pandemic preparedness strategies through vaccination. We assessed the breadth of the heterologous neutralizing antibody responses against the 3rd and 5th wave A(H7N9) viruses using the 1st wave vaccine sera from 4 vaccine groups: 1. inactivated vaccine with 2.8 μg hemagglutinin (HA)/dose + AS03_A_; 2. inactivated vaccine with 5.75 μg HA/dose + AS03_A_; 3. inactivated vaccine with 11.5 μg HA/dose + MF59; and 4. recombinant virus like particle (VLP) vaccine with 15 μg HA/dose + ISCOMATRIX™. Vaccine group 1 had the highest antibody responses to the vaccine virus and the 3rd/5th wave drifted viruses. Notably, the relative levels of cross-reactivity to the drifted viruses as measured by the antibody GMT ratios to the 5th wave viruses were similar across all 4 vaccine groups. The 1st wave vaccines induced robust responses to the 3rd and Pearl River Delta lineage 5th wave viruses but lower cross-reactivity to the highly pathogenic 5th wave A(H7N9) virus. The population in the United States was largely immunologically naive to the A(H7N9) HA. Seasonal vaccination induced cross-reactive neuraminidase inhibition and binding antibodies to N9, but minimal cross-reactive antibody-dependent cell-mediated cytotoxicity (ADCC) antibodies to A(H7N9).

## 1. Introduction

Pandemics can occur when novel viruses emerge and spread efficiently among human population that has little or no pre-existing immunity. As we are combating the current unprecedented coronavirus disease 2019 (COVID-19) pandemic caused by the severe acute respiratory syndrome coronavirus 2 (SARS-CoV-2), it is imperative that we continue to monitor the pandemic risk of other respiratory viruses, notably influenza. Over the past 100 years, influenza viruses have caused four global pandemics. Despite increased surveillance efforts and poultry vaccination programs, novel zoonotic influenza virus infections in humans continue to occur and remain a public health threat. Influenza A(H7N9) viruses first emerged in March 2013 in China. Between 2013 and 2017, epidemics of A(H7N9) zoonotic human infections have occurred as distinct waves. The majority of human infections were zoonotic, as a result of direct contact with birds from live bird markets [1]. Although most cases were concentrated in China, in 2015, two A(H7N9) cases were detected in Canada following travel to China during the 3rd epidemic wave [2]. The 5th wave that occurred from October 2016 through September 2017 was the largest and most severe wave to date [3]. The 5th wave viruses diverged into two genetically distant groups referred to as the Yangtze River Delta (YRD) lineage and Pearl River Delta (PRD) lineage. The A(H7N9) viruses that spread in the first four waves were low pathogenic avian influenza (LPAI) viruses, while highly pathogenic avian influenza (HPAI) A(H7N9) emerged and circulated widely during the 5th wave. Although there is no evidence of sustained human-to-human transmission, A(H7N9) caused considerable mortality and morbidity. As of May 2022, a total of 1568 confirmed A(H7N9) human infections with 616 deaths have been reported, with a case fatality ratio (CFR) of 39%; the CFR from the 5th wave HPAI viruses was even higher at approximately 50% [4,5]. The Center for Disease Control and Prevention (CDC)’s Influenza Risk Assessment Tool (IRAT) ranked A(H7N9) viruses as having a high potential to cause a pandemic [6].

Host immunity is a critical parameter in assessing population susceptibility to influenza virus infections. Studies from an A(H7N9)-affected area in China reported that poultry workers who were exposed to A(H7N9) had a low seroconversion rate (6.3%) and there was no sero-positivity in the general population in the region as measured by hemagglutination inhibition (HI) titers. Moreover, survivors of A(H7N9) infection had a higher percentage of HI antibody sero-positivity (65.8%) than did fatal cases (28.6%), suggesting HI antibodies contributed to protective immunity [7].

Vaccination is the most effective prophylactic measure available to lessen morbidity and mortality from influenza virus infections. The World Health Organization (WHO) has recommended several Candidate Vaccine Viruses (CVVs) to be developed against A(H7N9) since its emergence in humans [8]. Several candidate A(H7N9) vaccines have been developed based on different technology platforms and evaluated in clinical trials, including inactivated vaccines adjuvanted with AS03_A_ and MF59, recombinant virus-like particle (VLP) vaccines, recombinant protein vaccines and mRNA vaccines [9,10,11,12,13]. Most of these vaccines were based on the 1st wave virus antigens. In the United States (US), pre-pandemic A(H7N9) vaccine antigens and adjuvants have been incorporated in the National Pre-Pandemic Influenza Vaccine Stockpiles [11]. Yet, emerging strains that could cause a pandemic are likely to be antigenically distant from the vaccines that are currently available and stockpiled. Continued efforts are needed to assess pre-pandemic vaccines available in the stockpile for the levels of the heterologous cross-reactive immune responses against newly emerging viruses.

In this study, we evaluated the breadth of the heterologous cross-reactive neutralizing antibody responses against the 3rd and 5th wave A(H7N9) viruses following vaccination with the 1st wave antigens using sera collected from 4 clinical trials representing 3 vaccine technology platforms (inactivated vaccines adjuvanted with two oil-in-water emulsions, AS03_A_ or MF59, and recombinant VLP adjuvanted with a saponin nanoparticle, ISCOMATRIX™) and 4 antigen/adjuvant combinations. We also assessed the population immunity against A(H7N9) viruses in the US. Lastly, we evaluated cross-reactive antibody responses to A(H7N9) viruses following seasonal vaccination in various age groups using expanded immunological measurements including neutralizing antibodies, neuraminidase (NA) inhibition antibodies and antibody-dependent cell-mediated cytotoxicity (ADCC) antibody responses.

## 2. Materials and Methods

### 2.1. A(H7N9) Clinical Trial Vaccine Sera

A(H7N9) vaccine sera were collected from healthy adults enrolled in 4 clinical trials: 1 trial sponsored by GlaxoSmithKline (GSK) Vaccines (ClinicalTrials.gov identifier: NCT01999842, accessed 31 May 2022), 2 trials sponsored by the National Institute of Allergy and Infectious Diseases (NIAID) (ClinicalTrials.gov: NCT01942265 and NCT01938742), and 1 trial sponsored by Novavax Inc (ClinicalTrials.gov identifier: NCT01897701). Stored sera from the trials were used in the current study. From each clinical trial, a subset of sera was selected by the sponsors from strong responders (achieved seroconversion by HI to CVV) in the target vaccine arms based on the original immunogenicity evaluations of the trials (Table 1). In the trial sponsored by GSK (clinicaltrials.gov identifier: NCT01999842), sera were collected from 25 adults who received two doses of inactivated A/Shanghai/2/2013 vaccine adjuvanted with AS03_A_ [14], an oil-in-water emulsion containing α-tocopherol and squalene. The actual vaccine formulation was determined to be 2.78 μg hemagglutinin (HA)/dose (initially targeted at 3.75 μg HA/dose) by the single radial immunodiffusion (SRID) assay [15]. In the first clinical trial sponsored by NIAID (clinicaltrials.gov identifier: NCT01942265), sera were collected from 30 adults who received two doses of inactivated A/Shanghai/2/2013 vaccines also adjuvanted with AS03_A_. The vaccine formulation was determined to be 5.75 µg HA/dose (with the original target at 3.75 μg/dose) by SRID [9]. In the second clinical trial sponsored by NIAID (clinicaltrial.gov: NCT01938742), sera were collected from 30 adults who received 2 doses of inactivated A/Shanghai/2/2013 vaccine adjuvanted with MF59, an oil-in-water emulsion containing squalene [10]. The vaccine formulation was determined to be 11.5 μg HA/dose by SRID (initially targeted at 7.5 μg HA/dose). In the clinical trial from Novavax (clinicaltrial.gov identifier: NCT01897701) [13], sera were collected from 27 adults who received 2 doses of recombinant VLP vaccine based on A/Anhui/1/2013 A(H7N9), which included A/Anhui/1/2013 HA and NA and, to support the formation of particles, A/Indonesia/05/2005 A(H5N1) matrix protein. The VLPs were formulated at 15 µg HA/dose adjuvanted with ISCOMATRIX™ (CSL Behring LLC, King of Prussia, PA, USA) [15].

In all 4 vaccine groups, vaccines were administered with 2 doses 21 days apart. Paired sera were collected before the first dose and 21–28 days after the second dose. From the GSK trial with inactivated vaccine formulated at 2.8 μg HA/dose with AS03_A_, we also assessed the addition time point at 180 days post-the first vaccination from the same participants. The study was approved by Human Subject Research Determination Review Board of CDC.

### 2.2. Seasonal Influenza Vaccine Sera and Population Immunity Sera

Anonymous influenza seasonal vaccination sera were collected pre-vaccination and 28 days post-vaccination from 3 age groups: children (6–35 months), adults (18–49 years) and elderly (≥65 years) who received the 2012–2013 and 2015–2016 Northern Hemisphere egg-based seasonal trivalent inactivated influenza vaccines (IIV3).

To assess the population immunity to A(H7N9) viruses in the US, 900 sera from healthy individuals aged 6–102 years collected in 2010 by the National Health and Nutrition Examination Survey (NHANES) were used in the study. NHANES sera collection is a population-based survey conducted nationwide using a complex, multistage, probability sample design to select participants representative of the civilian, non-institutionalized US population. Sera were stratified by nine age groups: 6–11, 12–19, 20–29, 30–39, 40–49, 50–59, 60–69, 70–79 and ≥80 (in years). The study was approved by Human Subject Research Determination Review Board of CDC.

### 2.3. Influenza Viruses and Sequence Analysis

Six wild-type A(H7N9) viruses were used in the study, two from the 1st epidemic wave, A/Anhui/1/2013 (AH1) and A/Shanghai/2/2013 (SH2); one from the 3rd wave: A/British Columbia/1/2015 (BC1), three from the 5th wave: A/Hong Kong/61/2016 (HK61, Pearl River Delta lineage, PRD), A/Hong Kong/125/2017 (HK125, Yangtze River Delta lineage, YRD), and a HPAI A(H7N9) virus A/Guangdong/17SF003/16 (GD17SF003) also from the YRD lineage. Seasonal A(H3N2) viruses A/Victoria/361/2011, A/Perth16/2009 and A/Switzerland/9715293/2013 were also used. Viruses were propagated in 10–11 day old embryonated eggs. All studies using A(H7N9) viruses were conducted in Biosafety level 3 Enhanced (BSL3E) laboratories, and studies using seasonal viruses were conducted in Biosafety Level 2 (BSL2) laboratories.

Virus stocks used in the study were sequenced and HA sequences were analyzed using the BioEdit software (https://bioedit.software.informer.com, accessed on 26 October 2022).

### 2.4. Hemagglutination Inhibition (HI) Assays

HI assays for A(H3N2) viruses were performed using 0.5% turkey erythrocytes [16]. HI assays to detect antibody responses to H7 viruses were performed with a modified HI assay using horse erythrocytes as previously described [17]. In brief, sera were heat inactivated for 30 min at 56 °C and then tested for non-specific agglutinins and adsorbed with horse erythrocytes as needed. Sera were then treated with receptor-destroying enzyme for 18–20 h at 37 °C to remove any non-specific inhibitors that may have been introduced during hemadsorption, followed by heat inactivation prior to the HI assay. Sera were serially diluted two-fold and incubated for 30 min with 4 hemagglutination units per 25 µL of virus, and then incubated with 1% horse erythrocytes for 60 min. HI titer was defined as the reciprocal of the last dilution of serum that completely inhibited hemagglutination. Antibody titers <10 (initial dilution) were reported as 5.

### 2.5. Microneutralization (MN) Assays

MN assays were performed as previously described [16]. Heat inactivated human sera were serially diluted 2-fold and incubated with one hundred 50% tissue culture infection dose (TCID_50_) of influenza viruses. The virus-sera mixture was used to infect 1.5 × 10^4^/well Madin-Darby canine kidney cells and incubated overnight. The plates were fixed with cold 80% acetone and the presence of viral nucleoprotein was quantified by enzyme-linked immunosorbent assay (ELISA). Microneutralization titers were defined as the reciprocal of the highest serum dilution that showed 50% neutralization. Antibody titers <10 (initial dilution) were reported as 5.

### 2.6. Enzyme-Linked Lectin Assay (ELLA)

Neuraminidase inhibition antibodies (NAI) were detected using ELLA assay [18]. Reassortant influenza viruses with a mismatched H6 HA (H6 HA from A/turkey/California/Benn/1973 or A/turkey/Massachusetts/3740/1975) and target NA were used: H6N9 SH2 (N9 from SH2), H6N9 AH1 (N9 from AH/1); H6N1CA07 (N1 from A/California/07/2009), H6N2Vic361 (N2 from A/Victoria/361/2011). Briefly, sera were first heat inactivated. Serial two-fold diluted sera were then incubated with H6NA reassortant viruses in 96-well plates coated with fetuin for 16–18 h. Horse radish peroxidase (HRP)-labeled peanut agglutinin (lectin) was added to the wells and incubated for 2 h, followed by washing and addition of substrate to reveal enzymatic cleavage of fetuin by viral NA. NAI titers were calculated as the reciprocal of the highest dilution with at least 50% neuraminidase activity inhibition.

### 2.7. Neuraminidase ELISA

Nickel plates were coated overnight at 4 °C with his-tag recombinant NA at 200 ng/well, plates were then washed and blocked with 5% milk and 0.1% Tween20 in Phosphate buffer saline for one hour and washed again. Serial diluted sera were transferred to each well (100 uL/well) and incubated at room temperature for one hour. After 3 washes, HRP conjugated goat anti-human IgG was added and incubated for one hour. The reactivity was revealed by TMB substrate and read at an absorbance of 450 nm with a spectrophotometer.

### 2.8. Antibody-Dependent Cell-Mediated Cytotoxicity (ADCC) Natural Killer (NK) Cell Activation Assay

Influenza HA-specific ADCC-mediating antibodies were quantified using an ADCC-NK cell activation assay as described previously [19,20]. Two hundred nanograms per well of full-length, trimeric, recombinant A(H3N2) or A(H7N9) HA proteins were used as antigens. Sera were heat-inactivated and serially diluted starting with 1:40 dilution. The plates were then washed. Human NK cell line expressing high-affinity (158 *v*/*v*) FcγRIIIa receptor was mixed with PE-conjugated mouse anti-human CD107a. Following incubation and washing, the percentages of CD107a expression on NK cells were determined on a flow cytometer using a high throughput sampler. The ADCC antibody titers were defined as the highest serum dilution that achieved 3% threshold of CD107a + NK cells.

### 2.9. Statistical Analysis

Geometric mean antibody titers (GMTs) and 95% confidence intervals (CIs) were calculated. Seroconversion was defined as ≥4-fold rise in titers between pre- and post-vaccination with a post-vaccination titer ≥40. Seroconversion rate (SCR) is the proportion of the participants that seroconverted in the current study. Seroprotection rate (SPR) is the proportion (%) of the current study participants that achieved titers ≥40 by HI or MN [17,21,22]. Post-vaccination GMT ratios were calculated as “post-vaccination GMT to the heterologous virus/post-vaccination GMT to the homologous vaccine virus”. Sero-prevalence analysis was performed using SUDAAN program (RTI international) with standard survey methods using sample weights to account for the sampling design and oversampling of certain populations. All other statistical analysis were calculated using SAS 9.4 (SAS Institute) and Prism 8 (Graphpad).

## 3. Results

### 3.1. Breadth of the HI and Neutralizing Antibody Responses Elicited by the 1st Wave AS03_A_ and MF59 Adjuvanted Inactivated Vaccines and ISCOMATRIX Adjuvanted Recombinant VLP Vaccine

Antigenic characterization using ferret antisera showed that the two 5th wave viruses in the YRD lineage are antigenically drifted from the 1st wave virus A/Anhui/1/2013 (≥8 fold reduction in MN titer); the HPAI YRD lineage virus, A/Guangdong/17SF003/2016, is antigenically the most distant (16 fold reduction in MN titer, Supplementary Table S1) from the 1st wave virus. Sequence analysis of A(H7N9) viruses used in the study showed that the YRD lineage 5th wave virus A/Guangdong/17SF003/2016 has the highest number of amino acid differences in the viral HA compared to the HA of the 1st wave virus; this virus also has the multi-basic amino acid insertion in the cleavage site of the HA, representing the molecular signature of a HPAI virus (Supplementary Table S2).

**Figure 1:**
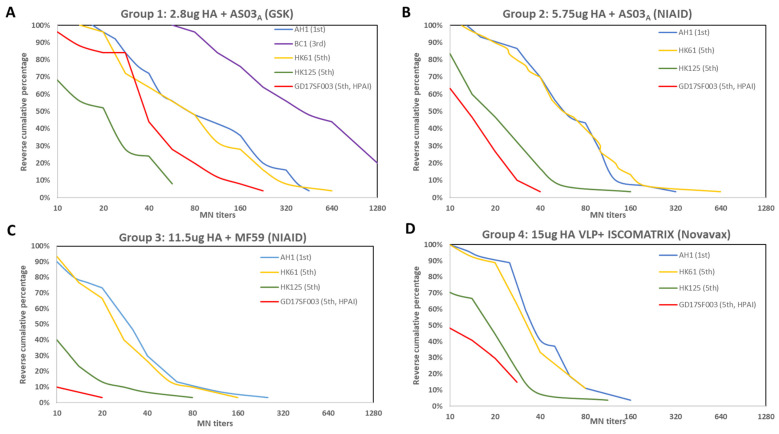
Reverse cumulative percentages of MN antibody responses to homologous and heterologous A(H7N9) viruses following A(H7N9) vaccination from 4 vaccine groups. Time points shown are 21–28 days post-2nd dose. (**A**) Vaccine group 1 received inactivated vaccines formulated at 2.8 μg HA/dose with AS03_A_ adjuvant from the GSK trial; (**B**) Vaccine group 2 received inactivated vaccine formulated at 5.75 μg HA/dose adjuvanted with AS03_A_ from the NIAID sponsored trial; (**C**) vaccine group 3 received inactivated vaccine formulated with 11.5 μg HA/dose adjuvanted with MF59 from the NIAID sponsored trial; (**D**) vaccine group 4 received VLP formulated at 15 μg HA/dose with ISCOMATRIX from the Novavax trial. AH1: A/Anhui/1/2013; HK61: A/Hong Kong/61/2016, HK125: A/Hong Kong/125/2017; GD17SF003: A/Guangdong/17SF003/2016.

Next, we evaluated vaccine sera from 4 vaccine groups immunized with 4 antigen dose/adjuvants combinations from 3 vaccine technology platforms by both HI and MN assays (Table 1): group 1. 2.8 μg HA/dose + AS03_A_ (GSK); group 2. 5.75 μg HA/dose + AS03_A_ (NIAID); group 3. 11.5 μg HA/dose + MF59 (NIAID), and group 4. 15 μg HA VLP/dose + ISCOMATRIX adjuvant (Novavax). Prior to vaccination, all participants were immunologically naive to A(H7N9) viruses (baseline titers <10). Following two doses of the 1st wave vaccines, participants from the vaccine group 1 who received 2.8 μg HA/dose + AS03_A_ had the highest antibody responses to the homologous 1st wave vaccine virus (MN GMT 95% CI: 77 {51–117}; participants from vaccine group 3 who received 11.5 μg HA/dose + MF59 had the lowest antibody responses to the homologous 1st wave wild type vaccine virus (MN GMT 95% CI: 25 {18–34}) (Table 1). In all 4 vaccine groups, the highest antibody responses were to the homologous wild type vaccine virus (A/Anhui/1/2013) (Table 1 and Figure 1), in one case (group 1 from GSK {2.8 μg HA/dose + AS03_A_}), a 3rd wave virus A/British Columbia/1/2015 was also tested and had the highest post-vaccination titers (MN GMT 95%CI: 329 {216–502}) (Table 1, Figure 1), suggesting that the 1st wave vaccine can provide strong protection against the 3rd wave virus representing the viruses isolated from the first imported North American A(H7N9) human cases [2].

**Figure 2:**
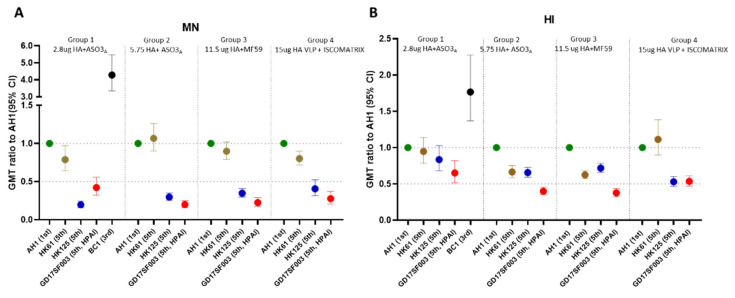
Post-vaccination GMT ratios of the heterologous MN and HI responses from the 3rd and 5th wave viruses compared to the 1st wave homologous A(H7N9) wild type vaccine viruses. Time points shown are 21–28 days post-2nd dose. GMT ratios were calculated as “post-vaccination GMT to the heterologous virus/post-vaccination GMT to the homologous vaccine virus”. (**A**) MN GMT ratios; (**B**) HI GMT ratios in 4 vaccine groups. GMT ratios with 95% confidence interval (95% CI) per vaccine group are shown.

Participants from all 4 vaccine groups also mounted various levels of cross-reactive antibody responses to the 5th wave viruses (Table 1), illustrated by the reverse cumulative percentage analysis of post-vaccination MN antibodies (Figure 1). Next, to quantify the relative level of cross-reactive responses, we used post-vaccination GMT ratio as a proxy (Figure 2). In all 4 vaccine groups, post-vaccination MN GMT ratios were the highest to the PRD lineage 5th wave virus (MN GMT ratio 0.79–1.06, HI GMT ratio: 0.62–1.11 against A/Hong Kong/61/2016), and the lowest to the HPAI YRD lineage 5th wave virus (MN GMT ratio: 0.2–0.42, HI GMT ratio: 0.38–0.65 against A/Guangdong/17SF003/2016) (Figure 2). Interestingly, the trend of the post-vaccination neutralizing antibody GMT ratios to each of the 5th wave viruses were similar across all 4 vaccine groups (Figure 2A). Participants in group 1 who received 2.8 μg HA/dose + AS03_A_ had the highest heterologous antibody responses, achieved 36% seroconversion and 44% SPR in both HI and neutralizing antibodies against the HPAI 5th wave virus at 42 days post-vaccination (21 days post-second dose) (Table 1).

**Figure 3:**
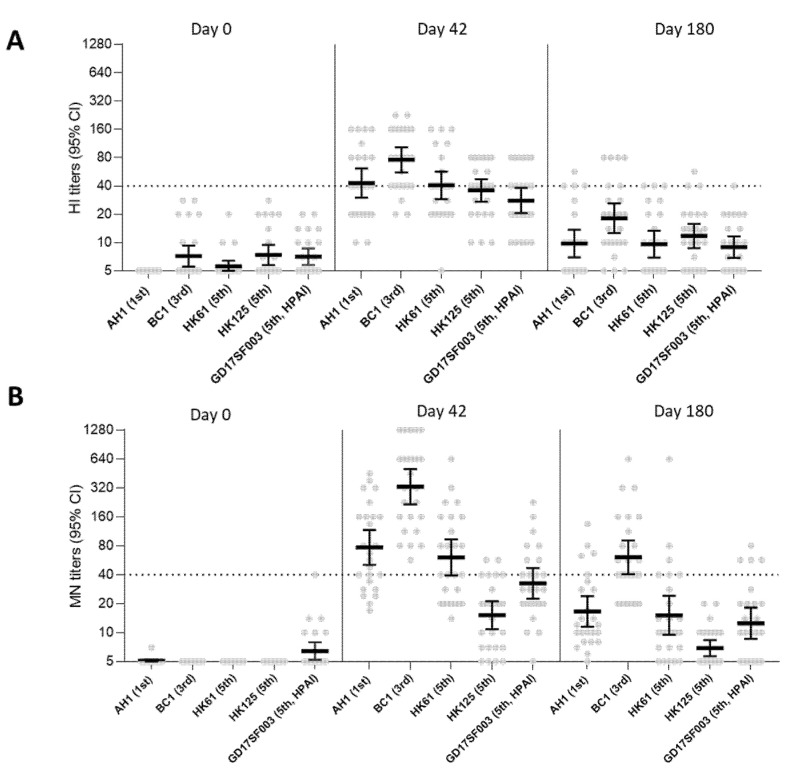
A(H7N9) HI and MN antibody responses at 0, 42, and 180 days post-1st dose following vaccination with 2.8 μg HA/dose + AS03_A_ in vaccine group 1. (**A**) HI antibody responses and (**B**) MN antibody responses to homologous and heterologous A(H7N9) viruses following vaccination. AH1: A/Anhui/1/2013; BC1: A/British Columbia/1/2015; HK61: A/Hong Kong/61/2016, HK125: A/Hong Kong/125/2017; GD17SF003: A/Guangdong/17SF003/2016. Dots in the scatter plots represents individual titers. GMTs with 95% confidence interval (CI) were shown. Dashed line denotes a titer of 40.

We then assessed antibody waning at 180 days post-1st dose in participants from group 1 who received 2.8 μg HA/dose adjuvanted with AS03_A_ (Figure 3)_._ At 6 months after the 1st dose, HI and neutralizing antibodies to both homologous and heterologous A(H7N9) viruses waned to almost baseline levels (GMT < 20) for most viruses, except that MN titers to the 3rd wave A(H7N9) virus (which had the highest post-vaccination titers) remained detectable at GMT > 40.

**Figure 4:**
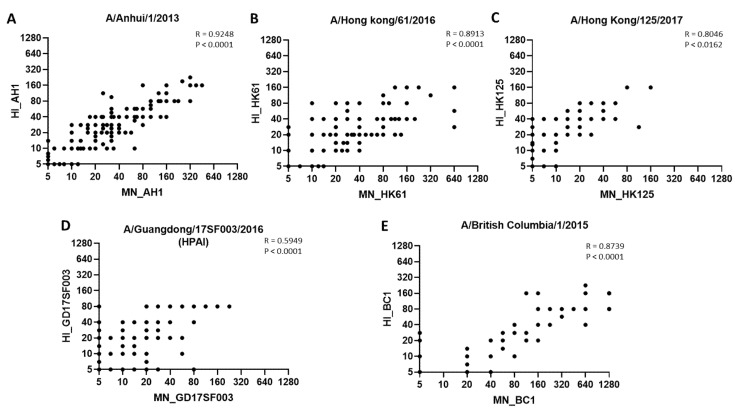
**Correlation between HI and MN antibody titers to 1st, 3rd and 5th wave A(H7N9) viruses.** Spearman correlation between HI and MN antibody titers to 5 A(H7N9) viruses. (**A**) A/Anhui/1/2013 (AH1); (**B**) A/Hong Kong/61/2016 (HK61); (**C**) A/Hong Kong/125/2017 (HK125); (**D**) A/Guangdong/17SF003/2016 (GD17SF003; (**E**) A/British Columbia/1/2015 (BC1). Dots represent titers to corresponding viruses.

Lastly, post-vaccination HI and MN titers to A(H7N9) viruses correlated well; spearman correlation R values ranged from 0.5949 to 0.9248 for all 5 A(H7N9) viruses (Figure 4).

### 3.2. No Pre-Existing Neutralizing Antibodies to HA of A (H7N9) Viruses in the US Population

Next, we evaluated population immunity to the 1st wave A(H7N9) viruses in 9 age groups (6–11, 12–19, 20–29, 30–39, 40–49, 50–59, 60–69, 70–79, ≥80 years) as measured by HI and neutralization antibodies to A(H7N9). No cross-reactive HI and neutralization antibodies were detected to A(H7N9) HA with 0% SPR in almost all 9 age groups (6–102 years old, *n* = 900), indicating that the US population is largely immunologically naive to the HA of A(H7N9) viruses. Because H7 and H3 are both group 2 influenza viruses, we also included a seasonal A(H3N2) virus representing the circulating A(H3N2) virus at the time of the sera collection in the analysis as a control. In contrast to A(H7N9) viruses, all age groups had pre-exiting antibodies to this seasonal A(H3N2) virus, demonstrating an age-related pattern (Table 2 and Supplementary Figure S1).

### 3.3. Seasonal Influenza Vaccination Elicited Heterologous Cross-Reactive NAI Antibodies, but Not HI, MN and ADCC Antibody Responses to A(H7N9)

We then evaluated whether seasonal influenza vaccination can induce cross-reactive antibodies to A(H7N9) viruses, we expanded our analysis to measure both HA-targeting neutralizing antibodies (by HI and MN) and other functional antibodies including NAI and ADCC antibodies. Seasonal vaccination with 2012–2013 IIV3 did not induce any cross-reactive HI or MN antibodies to both 1st wave A(H7N9) viruses tested (post-vaccination GMT by HI and MN: 5 {0}, 0% SPR to both AH1 and SH2) in children (6–35 months), adults (18–49 years) or elderly (≥65 years) (Table 3).

Surprisingly, a rise of NAI antibodies to N9 was detected in all 3 age groups that received 2012–2013 seasonal IIV3 (Table 3). Adults (18–49 years) had the highest NAI antibody fold rise to N9 (57–70% ≥4 fold rise to H6N9_SH2_ and H6N9_AH1_), whereas 17% elderly (≥65 years) and 20% children had ≥4 fold rise to H6N9_SH2_. To verify that the NA antibody rise detected was not H6NA reassortant virus dependent, we tested two reassortant N9 viruses bearing different strains of H6 and N9 (H6_A/turkey/Massachusetts/3740/1975_N9_AH1_ and H6_A/turkey/California/BENN/1973_N9 _SH2_) with adult sera. Similar NA antibody rises were detected by both H6NA reassortant viruses (Table 3). To further confirm the increase of NA antibodies following seasonal IIV3 vaccination, we then used ELISA to measure NA binding antibodies. Increased levels of NA binding antibodies to N9 were detected in all 3 age groups, 24% of the pediatric group, 27% of the adult group and 7% of the elderly had ≥ 2 fold rise in NA-binding antibodies to N9 post IIV3 vaccination (Table 3). Notably, NA binding antibodies had much higher titers than NAI titers in both pre- and post-vaccination sera (Table 3) but tended to manifest more modest increases than NAI antibodies. In contrast to human sera, antigenic characterization of NAs by ferret antisera demonstrated that N9 NAs from A(H7N9) were antigenically distinct from N1 and N2 in both ELLA and ELISA assays (Supplementary Tables S3 and S4), suggesting human sera have much broader N9 cross-reactivity than ferret sera which may reflect complex exposure to influenza in humans overtime.

Lastly, we assessed whether seasonal vaccination could induce cross-reactive HA-mediated ADCC antibody responses to A(H7N9). Although vaccination with 2015–16 IIV3 in adults induced robust ADCC antibodies to homologous A(H3N2) vaccine virus (61% ≥ 4 fold rise), it induced low cross-reactive ADCC antibodies to A(H7N9); only 4% (N = 1) of participants achieved ≥ 4 fold rise in H7-mediated ADCC antibodies to A/Shanghai/2/2013 (Table 4).

## 4. Discussion

Influenza viruses continue to pose a serious pandemic threat. As we are learning from the current unprecedented COVID-19 pandemic, vaccination has been proven to be the essential public health measure to combat the global pandemic [23]. The COVID-19 pandemic has also illustrated the gaps and challenges in the current pandemic preparedness strategies; a pandemic can be prolonged, and even during the same pandemic, multiple antigenically distinct new variants can rapidly emerge that can evade infection- or vaccine-induced immunity. Thus, vaccine technology platforms and vaccination strategies that can offer broad, long-lasting immunity and improved efficacy against heterologous emerging new variants are highly desirable.

Inactivated A(H7N9) vaccines alone induce poor immune responses in immunologically naïve populations; currently, potent adjuvants and 2 dose vaccine regimens are needed to provide sufficient immunogenicity against A(H7N9) [14]. At the onset of a potential influenza pandemic, rapid availability of vaccines to the whole population will be critical. In contrast to the total lack of vaccines during the early stage of the current COVID-19 pandemic, several candidate A(H7N9) vaccines based on AS03_A_, MF59, VLP and even mRNA technologies have already been developed and evaluated in the clinical trials for immunogenicity [9,10,11,12,13,14] and could be used in a pandemic response. Moreover, A(H7N9) vaccines have been included in the US National Pre-pandemic Influenza Vaccine Stockpiles [11]. Here, we evaluated the breadth of the HI and MN antibody responses using vaccine sera collected from 4 clinical trials with vaccines from 3 technology platforms: two are included in the US National Pre-pandemic Influenza Vaccine Stockpiles (inactivated antigens adjuvanted with AS03_A_ and MF59), and one newer technology platform based on VLP antigens with ISCOMATRIX™ adjuvant. Our results showed that vaccines containing the 1st wave A(H7N9) viruses can induce robust heterologous cross-reactivity against the 3rd wave and the PRD lineage 5th wave A(H7N9) viruses but low cross-reactivity against the YRD lineage 5th wave viruses especially the HPAI virus. Importantly, we found that the relative ratios of the heterologous neutralizing antibody responses induced to the drifted 5th wave viruses to the homologous wild type vaccine virus, as measured by post-vaccination GMT ratios, were similar across all 3 vaccine platforms (Figure 2A). Therefore, the absolute neutralizing antibody titers to the emerging heterologous strains (e.g., HPAI 5th wave viruses) were largely dependent upon the magnitude of the responses to the homologous vaccine strain (1st wave virus), suggesting it would be advantageous to improve the overall immunogenicity to the vaccine viruses. It should be noted that the 4 vaccine groups included in the current study were limited to the strong responders selected from 4 separate clinical trials, therefore our analysis was not focused on the direct comparison of the titers across these vaccine groups. Nevertheless, our results demonstrated that inactivated 1st wave vaccine antigens formulated at a low dose of 2.8 μg HA/dose adjuvanted with AS03_A_ still elicited some cross-reactive heterologous antibody responses to the antigenically drifted HPAI 5th wave virus and achieved 44% SPR in neutralizing and HI titers to this virus at 42 days post-1st dose vaccination (21 days post-2nd dose). It is also encouraging that the newer vaccine platform based on recombinant VLP and a saponin-based adjuvant also elicited heterologous antibody responses to the drifted 5th wave viruses.

Influenza viruses continue to undergo antigenic drift. In the event of an influenza pandemic caused by the A(H7N9) viruses, the pandemic strain is likely to be antigenically drifted from the current A(H7N9) vaccines. One scenario of A(H7N9) pandemic vaccination strategy is to rapidly deploy the existing vaccines as the priming dose as soon as possible at the onset of the pandemic, followed by a booster dose that is antigenically matched with the pandemic strains once it becomes available. In such a scenario, instead of requiring two doses, one booster dose with an antigenically matched pandemic strain could potentially elicit sufficient immunogenicity against the new pandemic viruses. Our previous study reported that heterologous prime-boost with different antigens could induce broader antibody responses than homologous prime-boost for HPAI A(H5N1) influenza viruses [17,22]. Furthermore, as demonstrated by the current ongoing COVID-19 pandemic, vaccines from different technology platforms could also be used together in a heterologous prime-boost regimen if authorized by FDA. The use of the existing A(H7N9) vaccines as a priming dose in a heterologous prime-boost regimen (with different antigens or even different technology platforms) could not only alleviate the demand for new vaccine doses during the pandemic, it may also provide broader immunity against drifted pandemic strains. The assessment of the breadth of the responses against the drifted strains from the current vaccine platforms therefore can provide insights to help identify the optimal vaccination strategies utilizing the existing vaccines and vaccine platforms for pandemic preparedness.

A pandemic can be prolonged; it is therefore also critical to understand the duration of immunity. Few A(H7N9) clinical trials have reported immune responses beyond 42 days post-1st dose of vaccination (21 days after the second dose). Here, we were able to include sera collected at 6 months post-1st dose of vaccination to assess the waning of cross-reactive antibodies. At 6 months following vaccination with 2.8 μg HA/dose + AS03_A_ (group 1), HI and neutralizing antibodies to both homologous vaccine virus and heterologous 5th wave viruses waned almost to the baseline level, suggesting one or more booster doses may be needed in the event of a prolonged pandemic.

Neutralizing antibodies targeting HAs on the surface of the influenza viruses, often measured as HI or MN titers, are considered correlated with protection against influenza infection [24]. An HI titer of 40 is considered to be associated with 50% reduction in influenza infection in adults [25,26,27]. We found that the population in the US is largely immunologically naïve to the HA of A(H7N9) viruses and with 0% SPR in both HI and neutralization antibody titers among the 900 people surveyed. This is also consistent with reports of other studies from China [7], Canada and elsewhere [2].

In addition to neutralizing antibodies, multiple immune mechanisms may contribute to immune protections from influenza infection. We previously reported that vaccination with inactivated 1st wave A(H7N9) virus adjuvanted with either AS03_A_ or MF59 elicited robust cross-reactive NAI antibodies and HA-specific ADCC antibody responses to the 5th wave A(H7N9) viruses [20]. The VLP vaccine with ISCOMATRIX, has also been shown to elicit strong N9 NAI responses [12]. Here, we assessed the level of cross-reactive NAI and ADCC antibody responses to A(H7N9) from seasonal vaccination. To our surprise, we detected robust increase of antibodies to N9 after vaccination with 2012–2013 seasonal IIV3 in adults both in functional NAI antibodies measured by ELLA, and in binding antibodies measured by ELISA. These results suggest that antibody immunity to N9 neuraminidase may exist in the population from cross-reactivity from seasonal vaccination. NAI antibodies can prevent virus egress, reduce viral shedding and lessen disease severity [28]. NAI antibody has been reported as an independent correlate of protection against influenza [29]. However, to date, the threshold of the NAI antibody that correlated with protection still remains to be determined, and the breadth of the neuraminidase antibody based cross-protection in humans is not well understood. The amount of neuraminidase in the US licensed seasonal vaccines is not determined and therefore the level of cross-reactive neuraminidase antibodies induced by seasonal vaccination may have a great deal of variability. Nevertheless, the role of neuraminidase antibodies in influenza protective immunity has been well recognized. Cross-reactive NAI antibodies to N9 in the population might confer partial protection in a A(H7N9) pandemic. In contrast to NAI antibody responses, we found that seasonal vaccination only induced minimum cross-reactive ADCC antibodies to H7 HA, suggesting that pre-existing cross-reactive H7-specific ADCC antibodies in the population is low.

Our study has limitations, first, for the A(H7N9) vaccine responses we only focused on the strong responders selected from the immunogenicity evaluation of the original study, because other participants that did not respond well to the vaccine virus likely had low cross-reactive responses to drifted viruses, thus, the level of heterologous cross-reactive responses from the strong responders represent a best case scenario. It also should be noted that in the original immunogenicity studies reverse genetics CVVs were used, in the current study we used wild type A(H7N9) vaccine viruses, therefore the titers may differ. Second, the 4 A(H7N9) vaccine groups were selected from 4 different clinical trials, a vaccine trial with a randomized comparison of optimized doses of the different vaccine technology platforms are not available for direct comparison of the responses.

## 5. Conclusions

Taken together, the continued antigenic evolution of influenza viruses with pandemic potential poses challenges for effective influenza pandemic mitigation through vaccination. Effective pandemic vaccines need to provide timely, broad, and long-lasting protection against antigenically drifted emerging variants. Immunological markers with demonstrated correlates of protection against influenza illness can be used to assess the vaccine immunogenicity and predictive efficacy against emerging drifted variants, and protective immunity. Such correlates of protection assessments can be expanded beyond the traditional HI and neutralization assays to include NAI and ADCC antibodies, and other relevant immunological measurements. Vaccines that can induce multiple arms of immune responses can not only prevent illness but may also interrupt transmission or alleviate the diseases severity to further mitigate the pandemic impact. Although the data presented in the current study do not represent a randomized comparison of the vaccines studied; nonetheless, they illustrate that both classical inactivated virus and recombinant technologies can address pandemic threats. Concerted efforts are needed to be better prepared for future influenza pandemics.

## Figures and Tables

**Table 1:** Homologous and heterologous HI and MN antibody responses to the 1st, 3rd and 5th wave A(H7N9) viruses following vaccination with the 1st wave vaccines in 4 vaccine groups based on 3 vaccine technology platforms and 4 antigen/adjuvant combinations.

#	Vaccine Groups	Clinical Trials NCT#	Clinical Trial Sponsor	Vaccine (Antigens)	Actual Vaccine Dose (μg HA per Dose)	Target Vaccine Dose (μg HA/Dose)	Adjuvant	N	Age (Median Range) in yrs	A(H7N9) Viruses			HI Titers				MN Titers			
										Strain Name	Epidemic Wave/Lineage	Pathogenicity	Pre-Vaccination	21–28 Days Post-2nd Dose			Pre-Vaccination	21–28 Days Post-2nd Dose		
													GMT (95% CI)	GMT 95% CI	% Seroconversion	% SPR	GMT (95% CI)	GMT 95% CI	% Seroconversion	% SPR
1	2.8 μg HA + ASO3_A_	NCT01999842	GSK	Inactivated A/Shanghai/2/2013	2.8	3.75	ASO3_A_	25	≥18	A/AH/1/2013	1st	LPAI	5	43 (30–61)	64	64	5 (5–5)	77 (51–117)	72	72
										A/BC/1/2015	3rd	LPAI	7 (6–9)	76 (56–103)	80	88	5	329 (216–502)	100	100
										A/HK/61/2016	5th/PRD	LPAI	6 (5–6)	41 (29–57)	64	64	5	61 (39–94)	64	64
										A/HK/125/2016	5th/YRD	LPAI	7 (6–9)	36 (27–47)	52	64	5	15 (11–21)	24	24
										A/GD/17SF003/2016	5th/YRD	HPAI	7 (6–9)	28 (20–38)	36	44	5 (5–8)	32 (23–47)	36	44
2	5.75 μg HA + ASO3_A_	NCT01942265	NIAID	inactivated (A/Shanghai/2/2013)	5.75	3.75	ASO3_A_	30	40 (19–59)	A/AH/1/2013	1st	LPAI	5 (5–6)	38 (29–49)	53	53	5 (–)	53 (40–71)	70	70
										A/HK/61/2016	5th/PRD	LPAI	5	25 (19–33)	40	40	5	57 (41–79)	70	70
										A/HK/125/2016	5th/YRD	LPAI	5	25 (19–32)	37	37	5 (5–5)	16 (12–21)	17	17
										A/GD/17SF003/2016	5th/YRD	HPAI	5	15 (12–20)	17	17	5 (5–6)	11 (8–13)	3	3
3	11.5 μg HA + MF59	NCT01938742	NIAID	inactivated (A/Shanghai/2/2013)	11.5	7.5	MF59	30	30 (21–58)	A/AH/1/2013	1st	LPAI	5	25 (19–32)	23	23	5	25 (18–34)	30	30
										A/HK/61/2016	5th/PRD	LPAI	5	15 (12–20)	10	10	5	22 (16–30)	27	27
										A/HK/125/2016	5th/YRD	LPAI	5	18 (13–23)	17	17	5	9 (7–11)	7	7
										A/GD/17SF003/2016	5th/YRD	HPAI	5	9 (7–12)	3	3	5 (5–5)	6 (5–6)	0	0
4	15 μg HA + ISCOMATRIX	NCT01897701	Novavax	recombinant VLP (A/Anui/1/2013)	15	NA	ISCO-MATRIX	27	33 (18–49)	A/AH/1/2013	1st	LPAI	5	40 (33–50)	67	67	5	35 (28–44)	41	41
										A/HK/61/2016	5th/PRD	LPAI	5	45 (38–53)	81	81	5	28 (23–35)	33	33
										A/HK/125/2016	5th/YRD	LPAI	5	21 (17–27)	26	26	5	14 (11–19)	7	7
										A/GD/17SF003/2016	5th/YRD	HPAI	5	22 (17–28)	22	22	5 (5–6)	10 (7–13)	0	0

PRD: Pearl River Delta; YRD: Yangtze River Delta. % seroconversion rate defined as the proportion of seroconversion. Seroconversion is defined as 4 fold rise from S1 to S2 with S2 ≥ 40. % SPR (percent seroprotection rate) defined as the proportion with titers ≥4.

**Table 2:** Seroprevalence of HI and MN antibodies to A(H7N9) and A(H3N2) viruses in the US population (2010) across 9 age groups (6–102 years, N = 900).

Age Groups (Age Range in Years)	Median Age (Years)	No of Subjects	HI				MN			
			A(H7N9) (AH1)		A(H3N2) (Perth16)		A(H7N9) (AH1)		A(H3N2) (Perth16)	
			GMT (95% CI)	SPR (%)	GMT (95% CI)	SPR (%)	GMT (95% CI)	SPR (%)	GMT (95% CI)	SPR (%)
6–11	9	100	5 (-)	0	34 (25–45)	55	5 (-)	0	99 (65–150)	73
12–19	15	100	5 (-)	0	19 (11–34)	32	5 (-)	0	59 (32–108)	59
20–29	24	100	5 (-)	0	9 (7–12)	13	5 (-)	0	50 (33–77)	54
30–39	34	100	5 (-)	0	7 (6–8)	5	5 (-)	0	21 (15–30)	34
40–49	44	100	5 (-)	0	8 (6–10)	8	5 (-)	0	22 (15–32)	29
50–59	54	100	5 (-)	0	12 (7–20)	19	5 (-)	0	33 (17–66)	46
60–69	62	100	5 (-)	0	11 (8–14)	19	5 (-)	0	32 (18–56)	42
70–79	73.5	100	5 (-)	0	14 (10–18)	27	5 (-)	0	49 (32–72)	61
≥80	83	100	5(5–6)	1	11 (9–15)	23	5 (-)	0	41 (30–57)	55

**Table 3:** HI, MN, NAI and NA binding antibody responses to A(H7N9) following seasonal IIV3 vaccination in children, adults and elderly.

Age Groups			6–35 Mos (Pediatrics)			18–49 Yrs (Adult)				≥ 65 Yrs (Elderly)		
**No.**			30			30				30		
	**Viruses**		**H3N2 _Vic361_**	**H7N9 _AH1_**	**H7N9 _SH2_**	**NT ^#^**	**H3N2 _Vic361_**	**H7N9 _AH1_**	**H7N9 _SH2_**	**H3N2 _Vic361_**	**H7N9 _AH1_**	**H7N9 _SH2_**
**HI**	**GMT**	Pre * (95% CI)	6 (5–6)	5 (-)	5 (-)	NT	16 (9–29)	5 (-)	5 (-)	34 (20–57)	5 (-)	5 (-)
		Post * (95% CI)	58 (35–95)	5 (-)	5 (-)	NT	145 (91–230)	5 (-)	5 (-)	94 (62–143)	5 (-)	5 (5–6)
	% with HI ≥ 40 Post *		73	0	0	NT	90	0	0	83	0	0
	**% ≥ 4 fold rise**		73% (22)	0	0	NT	63% (19)	0	0	27% (8)	0	0
	**Viruses**		**H3N2 _Vic361_**	**H7N9 _AH1_**	**H7N9 _SH2_**	**NT**	**H3N2 _Vic361_**	**H7N9 _AH1_**	**H7N9 _SH2_**	**H3N2 _Vic361_**	**H7N9 _AH1_**	**H7N9 _SH2_**
**MN**	**GMT**	Pre * (95% CI)	8 (5–11)	5 (-)	5 (-)	NT	34 (19–60)	5 (-)	5 (-)	88 (49–153)	5 (-)	5 (-)
		Post * (95% CI)	78 (47–130)	5 (-)	5 (-)	NT	298 (189–472)	5 (-)	5 (-)	240 (159–362)	5 (5–6)	5 (-)
	**% with MN ≥ 40 Post**		70	0	0	NT	97	0	0	90	0	0
	**% ≥ 4 fold rise (n)**		67% (20)	0	0	NT	57% (17)	0	0	30% (9)	0	0
**NAI functional antibodies (ELLA)**	**Viruses**		**H6N1 _CA07_**	**H6N9 _SH2_**	**NT**	**H6N1_CA07_**	**H6N2 _Vic 361_**	**H6N9_AH1_**	**H6N9_SH2_**	**H6N1 _CA07_**	**H6N9 _SH2_**	**NT**
	**GMT**	Pre * (95% CI)	6 (5–8)	5 (-)	NT	27 (16–45)	23 (17–32)	13 (10–18)	11 (8–15)	228 (135–388)	97 (68–138)	NT
		Post * (95% CI)	19 (12–29)	8(6–11)	NT	124 (77–200)	70 (56–87)	44 (32–61)	52 (33–81)	347 (211–570)	189 (122–292)	NT
	**% ≥2 fold rise % (N)**		63% (19)	30% (9)	NT	97% (29)	80% (24)	80% (24)	90% (27)	50% (15)	63% (19)	NT
	**% ≥4 fold rise %(N)**		50% (15)	20% (6)	NT	73% (22)	57% (17)	57% (17)	70% (21)	7% (2)	17% (5)	NT
**NA binding antibodies (ELISA)**	**rNAs**		**rN1 _CA07_**	**rN9 _AH1_**	**NT**	**rN1 _CA07_**	**NT**	**rN9 _AH1_**	**NT**	**rN1 _CA07_**	**rN9 _AH1_**	**NT**
	**GMT**	Pre * (95% CI)	4783 (3477–6581)	3676 (2589–5218)	NT	6475 (4590–9134)	NT	3469 (2751–4376)	NT	11,273 (7812–16,267)	3200 (2314–4425)	NT
		Post * (95% CI)	7558 (5781–9882)	4050 (2928–5603)	NT	12362 (8105–18856)	NT	4371 (3314–5765)	NT	13,878 (10,227–18,832)	3313 (2403–4568)	NT
	**% ≥2 fold rise %(N)**		52% (13)	24%(6)	NT	63% (19)	NT	27% (8)	NT	20% (6)	7% (2)	NT
	**% ≥4 fold rise %(N)**		16%(4)	0% (0)	NT	27% (8)	NT	20% (6)	NT	3% (1)	0% (0)	NT

* Pre and Post vaccination in the 2012–2013 influenza season. ^#^ NT: not tested; HI: hemagglutination inhibition, MN microneutralization, NAI: neuraminidase inhibition antibodies, IIV3, trivalent seasonal vaccination. AH1: A/Anhui/1/2013; SH2: A/Shanghai/2/2013; Vic361: A/Victoria/361/2011; CA07: A/California/07/2009.

**Table 4:** HA-mediated ADCC antibody responses to A(H7N9) and A(H3N2) viruses following seasonal IIV3 vaccination in adults.

Assay	Antigen	Subtype	GMT		% ≥4 Fold Rise %(N)
			Pre * (95% CI)	Post * (95% CI)	
MN	A/Switzerland/9715293/2013#	A(H3N2)	22 (12-43)	542 (364-806)	87% (20)
HI	A/Switzerland/9715293/2013#	A(H3N2)	16 (9-28)	330 (211-517)	91% (21)
ADCC	A/Switzerland/9715293/2013#	A(H3N2)	94 (59-152)	453 (331-619)	61% (14)
ADCC	A/Shanghai/2/2013	A(H7N9)	37 (24-58)	48 (30-76)	4% (1)

*: pre and post vaccination in 2015–2016. #: vaccine component. CI: confidence interval.

## Data Availability

The data supporting the finding of the study can be available from the corresponding author upon reasonable request.

## Supplementary Materials

The following supporting information can be downloaded at: https://www.mdpi.com/article/10.3390/vaccines10111856/s1, Table S1: Antigenic characterization of the 1st and 5th wave A(H7N9) viruses by microneutralization assay (MN) using ferret antisera; Table S2: Amino acid difference in HA1 of A(H7N9) viruses; Table S3: Antigenic characterization with N1, N2, and N9 NAI antibodies by ELLA using ferret antisera; Table S4: Antigenic characterization of neuraminidase binding antibodies by ELISA using ferret antisera and monoclonal antibodies; Figure S1: Population immunity measured by HI and MN titers to A(H7N9) and A(H3N2) viruses in the US population (2010).
