# Supplementary material for: Influenza A(H7N9) Pandemic Preparedness: Assessment of the Breadth of Heterologous Antibody Responses to Emerging Viruses from Multiple Pre-Pandemic Vaccines and Population Immunity
Source: Vaccines (Basel). 2022 Nov 1;10(11):1856. doi: 10.3390/vaccines10111856 (PMC9694415; doi:10.3390/vaccines10111856)
Supplement: Supplementary file 1 [file vaccines-10-01856-s001.zip › vaccines-1982990-supplementary.pdf]

Supplementary Material

Influenza A(H7N9) Pandemic Preparedness: Assessment of the Breadth of Heterologous Antibody Responses to Emerging Viruses from Multiple Pre-Pandemic Vaccines and Population Immunity

Min Z. Levine[1*] , Crystal Holiday[1], Yaohui Bai[1], Weimin Zhong[1], Feng Liu[1], Stacie Jefferson[1], F Liaini Gross[1], Wen-pin Tzeng[1], Louis Fries[2], Gale Smith[2], Philippe Boutet[3], Damien Friel[3], Bruce L. Innis[4#], Corey P. Mallett[4], C. Todd Davis[1], David E. Wentworth[1], Ian A. York[1], James Stevens[1], Jacqueline M. Katz[1], Terrence Tumpey[1]

1. Influenza Division, National Center for Immunization and Respiratory Diseases, Centers for Disease Control and Prevention, Atlanta, GA, USA.
2. Novavax, Inc. Gaithersburg, MD, USA
3. GSK, Wavre, Belgium
4. GSK, Rockville, MD, USA

***Corresponding author:** Min Z Levine, Influenza Division, National Center for Immunization and Respiratory Diseases, Centers for Disease Control and Prevention, MS-H17-5, 1600 Clifton Road, Atlanta, GA 30329, USA. E-mail: mlevine@cdc.gov; Phone: +1 404 639 3504

[#] **Current Addresses:** PATH, Seattle Washington, USA.

Supplementary Table S1: Antigenic characterization of the 1st and 5th wave A(H7N9) viruses by microneutralization assay (MN) using ferret antisera.

A(H7N9) viruses	Epidemic wave/lineage		Reference antisera to			
			A/Anhui/1/2013	A/Hong Kong/61/2016	A/Hong Kong/125/2017	A/Guangdong/17SF003/2016
A/Anhui/1/2013	1st	LPAI	**1280**	160	40	1280
A/Hong Kong/61/2016	5th/PRD	LPAI	640	**320**	40	1280
A/Hong Kong/125/2017	5th/YRD	LPAI	160	40	**80**	320
A/Guangdong/17SF003/2016	5th/YRD	HPAI	80	14	20	**640**

Supplementary Table S2. Amino acid difference in HA1 of A(H7N9) viruses.

Mature H7 HA	A/Anhui/01/2013	A/Shanghai/2/2013	A/British Columbia/1/2015	A/Hong Kong/61/2016	A/Hong Kong/125/2017	A/Guangdong/17SF003/2016 (HPAI)
37	N		S			
38	I					T
104	E			K		
112	A				T	P
118	S				N	N
125	A				V	V
130	R				K	
163	R					K
164	K					E
168	L		I	I	I	I
184	K		N			
217	L					Q
227	M			I	I	I
261	G					R
267	N			D		
282	N			K		
290	S		R			
317	I					V
320	G					R
321	-					K
322	-	-	-	-	-	R
323	-	-	-	-	-	T
324	-	-	-	-	-	A
Total number of amino acid differences		0	4	5	6	16

Note: Multi-basic cleavage site is shaded in grey. HA numbering is based on mature H7 HA.

Supplementary Table S3. Antigenic characterization with N1, N2, and N9 NAI antibodies by ELLA using ferret antisera

NA	Reference ferret antisera to			
antigen	H1N1 $_{CA07}$	H3N2 $_{Vic\ 361}$	H7N9 $_{SH2}$	H7N9 $_{AH1}$
H6N1 $_{CA07}$	**5120**	10	40	40
H6N2 $_{Vic\ 361}$	40	**5120**	40	40
H6N9 $_{SH2}$	20	20	**5120**	5120
H6N9 $_{AH1}$	20	10	2560	**1280**

Supplementary Table S4. Antigenic characterization of neuraminidase binding antibodies by ELISA using ferret antisera and monoclonal antibodies

Antigen	Ferret anti H1N1 $_{CA07}$	N1 Mab*	Ferret anti H7N9 $_{AH1}$	N9 Mab*
rN1 $_{CA07}$	**25600**	**400**	<100	<100
rN9 $_{AH1}$	<100	<100	**51200**	**102400**

*: Mab (monoclonal antibodies) to N1 and N9 were obtained from International Reagent Resource (https://www.internationalreagentresource.org/)

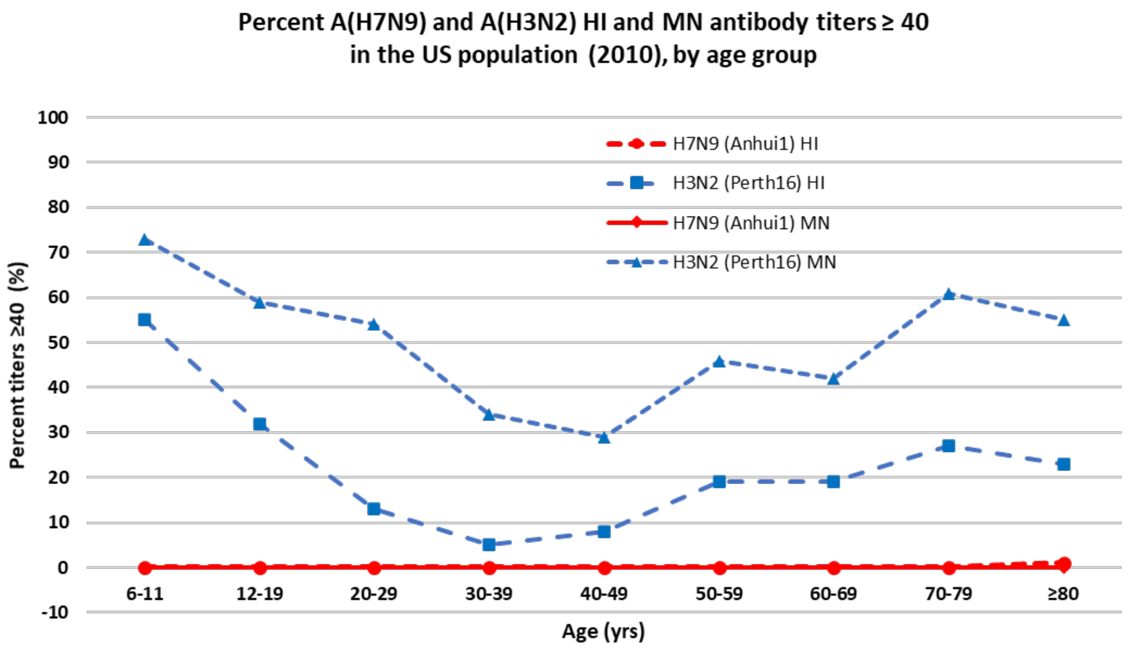

Supplementary Figure S1. Population immunity measured by HI and MN titers to A(H7N9) and A(H3N2) viruses in the US population (2010). NHANES 2010 sera were used in the analysis. Percent HI and MN titers ≥ 40 were graphed by age groups.
